# The Treatment of Syphilis

**Published:** 1871-10

**Authors:** 


### The Treatment of Syphilis.

Syphilis, says M. Bazin in a work recently issued, containing his clinical experience at the Hospital Saint Louis, Paris, is a disease which takes its place rightly by the side of scrofula, arthritism, and herpetism. Now, syphilis has but one specific remedy—mercury, and as we do not meet with mercury in any mineral water, it is necessary to have recourse to the "Pharmacopœia." An hospital surgeon, M. Depres, of Paris, has expressed himself as very hostile to this drug, and has almost taken up the fulminations of Broussais to stigmatise it. According to M. Depres, mercury is useless, and dangerous—useless, because Nature cures syphilis very well without Art intervening; dangerous, because it produces a crowd of disasterous effects which every one knows. Unfortunately, for

M. Depres, says Bazin, this doctrine is not tenable from any point of view. With regard to the action of Nature, clinical observation shows that it is only the benignant affections in syphilis which are cured without the assistance of Art. It is not the less certain, he says, that mercury acts not only on these affections, but also on the generality of syphilitic manifestations—that it has upon these an undeniably specific action. In reply to the argument as to the multiple accidents produced by mercury, Bazin admits these, but says that a good practitioner ought to know how to prevent and to correct these, whether by associating with mercury either chlorate of potash or iodide or bromide of potassium, or by making appeal to certain mineral springs, hydropathy, or other agents which are part of the specific treatment. He does not use the pills of Sedillot, because they salivate too rapidly. He prefers the pills of protoiodide of mercury, and prescribes Van Swieten's liquor in his hospital, which contains the thousandth part of its weight of corrosive sublimate. For ulcerated syphilides in the third period Bazin recommends a mixture of biniodide of mercury and iodide of potassium, and in children the same mixture. He uses mercury in all periods of syphilis, in the indurated chancre and in tertiary symptoms. In the second period Bazin does not admit in any way the distinction made by Diday between the benignant accidents which that doctor leaves without mercury and the malignant accidents which require it. Bazin says that slight secondary symptoms give no prognosis as to the gravity of tertiaries. When tertiary symptoms have arrived, some doctors give iodide of potassium and abandon mercury. This, according to Bazin, is a fault; they should be associated, not separated. In the fourth period of visceral lesions mercury is still indispensable, unless the patient has been salivated by it, in which case iodide and bromide of potassium are indicated, or sulphur. M. Bazin says iodide of potassium is not sufficiently powerful against the neoplasms of syphilis, and that the physician will not be certain to cure a patient attacked with ulcerated syphilides with iodide alone if the patient has not taken a mercurial course. On the other hand, iodide of potassium succeeds marvelously well when mercury has been given to the patient, even long before the appearance of these latter accidents. The formula he employs is 200 grammes of water to 6 of iodide of potassium. A tablespoonful for a dose, which gives about eight grains for a dose. But these doses should be quickly raised, and in two or three weeks amount to sixty or seventy-five grains a day, without going further.

Dr. Kraus, in the *Alg. Wien. Med. Zeit.* of 27th June, says that the French and English still use blue pill in syphilitic bubo, leeches, with purges, and compression by means of compressors. Ricord advised in bubos accompanying chancre, blister and calomel dressings; but Dr. Kraus considers such practice as *barbarous*. The first requisite in such cases is absolute rest, as well as attention to the ulcer which caused the bubo, which should neither be covered with strong astringents or strongly cauterised; cold bandages should be placed on the swelling and tincture of iodine painted on it; the skin over the swelling is to be rubbed thrice a day with a liniment composed of equal parts of tincture of iodine and galls and cold dressings after. In this way doubtless many favorable results are obtained. Some say that the good result is entirely attributable to rest and the application of cold bandages. If the resorption of the swelling cannot be effected by this treatment, and true fluctuation is felt at any point, the abscess should be punctured and softly emptied by pressing on from the periphery. The swelling is then dressed with cold dressing and painted with tincture of iodine or a plate of lead with a weight above it of half a pound laid on the parts. In order to prevent the dangerous entry of air into the abscess Zeissl puts many of his patients into sitz-baths, and punctures the abscess under water, then covers the parts with plaster of Paris, which prevents the entrance of air and exerts pressure on the parts. Should the opening close up after being punctured, it is opened again with the sound, or punctured afresh. In this way, in many cases, we succeed even in extending abscess, and when the skin is extensively reddened to a perfect cure. The advantages of this method over the opening by incision or cautery are indescribable, and we need only look at the ugly great scars which remain after these methods, and give, too, a predisposition to hernia, to see what an improvement this practice is. The chief advantage of puncture resides in the notable shortening of the period of treatment, as the cure takes place in two-thirds of the time it took after cautery. We must notice, that even an extended abscess and reddened skin are not always contra-indications against this method. We have only to make several punctures (in order to prevent the ulceration of the thinned integument) around the swelling and on the healthy integument. The sooner such punctures are made, the more likely we are to have fortunate results; but even in cases of long chronic bubos, full of pus, we should always try what puncture will accomplish, since when the puncture does not succeed, the incision can always be resorted to. The gangrene of the edges of the puncture, which often taken place when the puncture is made through very thin skin, is truly a very disagreeable event; but, even then, cure takes place much quicker and less painfully than it does after crucial incisions; besides which the other methods are often followed by severe results.—*The Doctor.*

